# Molecular determinants of ovarian cancer chemoresistance: new insights into an old conundrum

**DOI:** 10.1111/j.1749-6632.2012.06734.x

**Published:** 2012-10-10

**Authors:** Ahmed Y Ali, Lee Farrand, Ji Young Kim, Sanguine Byun, Jeong-Yong Suh, Hyong Joo Lee, Benjamin K Tsang

**Affiliations:** 1 Department of Cellular and Molecular Medicine, University of OttawaOttawa, Ontario, Canada; 2 Department of Chronic Disease Program, Ottawa Hospital Research InstituteOttawa, Ontario, Canada; 3 Department of Agricultural Biotechnology, College of Agriculture and Life Sciences, Seoul National UniversitySeoul, Republic of Korea; 4 Department of Obstetrics and Gynecology, University of OttawaOttawa, Ontario, Canada

**Keywords:** ovarian cancer chemoresistance, p53, PPM1D, PI3K/Akt, functional food compounds

## Abstract

Ovarian cancer is the most lethal gynecological malignancy. Cisplatin and its derivatives are first-line chemotherapeutics, and their resistance is a major hurdle in successful ovarian cancer treatment. Understanding the molecular dysregulation underlying chemoresistance is important for enhancing therapeutic outcome. Here, we review two established pathways in cancer chemoresistance. p53 is a major tumor suppressor regulating proliferation and apoptosis, and its mutation is a frequent event in human malignancies. The PI3K/Akt axis is a key oncogenic pathway regulating survival and tumorigenesis by controlling several tumor suppressors, including p53. The interplay between these pathways is well established, although the oncogenic phosphatase PPM1D adds a new layer to this intricate relationship and provides new insights into the processes determining cell fate. Inhibition of the PI3K/Akt pathway by functional food compounds as an adjunct to chemotherapeutics may tip the balance in favor of apoptosis rather than survival, enhancing therapeutic efficacy, and reducing side effects.

## Ovarian cancer chemoresistance

Ovarian cancer is the most lethal of all gynecological malignancies, primarily due to asymptomatic presentation of the disease and late diagnosis.^1^ The conventional course of therapy for ovarian cancer includes surgical debulking of the tumor mass followed by adjuvant chemotherapy. Cisplatin (*cis*-diamminedichloroplatinum (CDDP)) and its platinum derivatives are first-line chemotherapeutic agents in the treatment of ovarian cancer. CDDP induces apoptosis through irreversibly intercalating DNA through inter- and intrastrand DNA adducts, thereby inducing the DNA damage response and the activation of apoptotic machinery. Most patients are responsive to chemotherapy at first; however, recurrent ovarian tumors are more aggressive, metastasize to secondary target tissues, and acquire resistance to conventional chemotherapeutics. Drug resistance is a multifactorial problem and is characterized by acquired genetic mutations, which can lead to dysregulation of the balance between cellular survival pathways and apoptosis-regulating tumor suppressors, enhanced drug clearance and detoxification, and reduced drug efficacy due to an increase in DNA repair.^2^

## Ovarian cancer and p53

The tumor suppressor p53 is involved in the regulation of cellular proliferation and apoptosis through the control of several molecular pathways. p53 elicits its actions via transcription-dependent and transcription-independent mechanisms.^3^,^4^ We have shown that a functional p53 signaling pathway is necessary to sensitize cancer cells to DNA-damaging chemotherapeutic agents, such as CDDP.^3^,^5^–^11^ p53 is activated in response to genomic insults by the DNA damage sensors ataxia talengiectasia mutated protein (ATM) and ataxia talengiectasia and Rad3-related protein (ATR) and their downstream effectors checkpoint kinases 1 and 2 (Chk1 and Chk2). In turn, p53 maintains a sustained cell cycle arrest by upregulating the expression of the cyclin-dependent kinase inhibitor p21, which arrests the cell cycle at the G_1_ phase,^12^ and 14-3-3σ, which sequesters CDC25C phosphatase in the cytoplasm and promotes G_2_/M cycle arrest.^13^,^14^ Furthermore, p53 induces the expression of several proapoptotic proteins, which act on the mitochondria and cause the release of mitochondrial death proteins. These include p53 upregulated modulator of apoptosis (PUMA), NADPH oxidase activator (NOXA),^9^,^15^,^16^ Bcl-2 family members Bax and Bid,^17^,^18^ and apoptotic peptidase activating factor 1 (Apaf-1).^19^ p53 also upregulates the expression of the death receptors Fas, DR4, and DR5.^20^–^22^ These few examples of p53-regulated genes belong to a growing list that emphasizes the multifactorial role of the p53 network in tumor suppression. Mutations and/or functional inactivation of p53 are a hallmark of many human malignancies, including ovarian cancer.^23^,^24^

## Regulation of the DNA damage response and apoptosis by PPM1D

Protein phosphatase magnesium/manganese-dependent 1 D (PPM1D), also known as wild-type p53 inducible phosphatase (Wip1) and protein phosphatase type 2C delta (PP2Cδ), is a member of the type 2C phosphatase family, specifically belonging to the magnesium/manganese-dependent subfamily of PPM1 phosphatases. It was first identified as a p53-induced phosphatase in response to ultraviolet (UV) and ionizing radiation (IR).^25^–^27^ While PPM1D is induced by p53 in response to DNA damage, recent evidence has revealed that PPM1D is also regulated by several transcription factors, including cyclic AMP response element binding protein (CREB),^27^ NF-κB,^28^ estrogen receptor α (ERα),^29^ E2F1,^30^ and c-Jun.^31^ PPM1D preferentially dephosphorylates phosphoproteins containing SQ/TQ or TXY motifs.^25^,^32^–^34^

**Figure 1:**
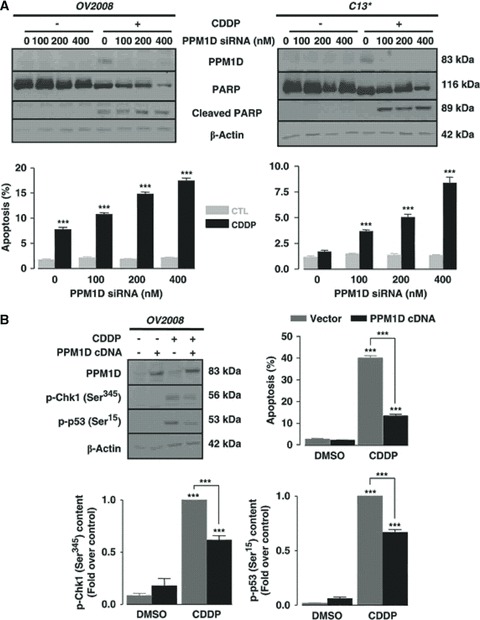
Involvement of PPM1D in the regulation of CDDP sensitivity in ovarian cancer (OVCA) cells. (A) siRNA-mediated PPM1D downregulation–sensitized C13* cells, as evident by apoptotic cell count and poly (ADP-ribose) polymerase (PARP) cleavage, and enhanced sensitivity of OV2008 to CDDP-induced apoptosis in a concentration-dependent manner. OV2008 and C13* cells were incubated with PPM1D siRNA or control siRNA (0–400, 24 h), treated with CDDP (left panel: 0–10 μM, 12 h; right panel: 0–10 μM, 24 h), and PPM1D, PARP, cleaved PARP, and β-actin contents and apoptosis were assessed (*n*= 4). (B) Overexpression of PPM1D in OV2008 significantly decreased CDDP-induced apoptosis (*P* < 0.001), which was associated with decreased p-Ser^345^-Chk1 and p-Ser^15^-p53 contents (*P* < 0.01). OV2008 cells were transfected (1 μg, 24 h) with PPM1D cDNA or empty pCMV6-XL5 vector, treated with CDDP (0–10 μM, 24 h), and PPM1D, p-Ser^345^-Chk1, p-Ser^15^-p53, β-actin contents, and apoptosis were assessed (*n*= 3). ***P < 0.001 (versus respective CTL). From Ref. 11.

Under normal conditions, PPM1D restores cellular homeostasis following DNA-damage by cooperating with p53 to induce G_2_/M cell cycle arrest, thereby allowing ample time for repair of the damaged DNA.^35^–^37^ However, PPM1D amplification and/or enhanced stabilization allow for sustained inhibition of DNA damage response proteins and numerous tumor suppressors. PPM1D overexpression has been implicated in a variety of human malignancies, including ovarian carcinoma, and its level is directly related to poor prognosis and reduced therapeutic outcome.^38^–^48^ PPM1D has been identified as a potent oncogene, enhancing mammary transformation in a breast cancer-susceptible animal model.^49^ Moreover, PPM1D-null mice exhibit a lower incidence of spontaneously occurring tumors^50^ and resistance to oncogene-induced transformation.^49^,^51^,^52^ The primary function of PPM1D in tumorigenesis is the attenuation of DNA damage and apoptotic responses following a genomic insult. This occurs through the regulation of the ATM/ATR and p53 pathways. ATM was found to be directly dephosphorylated by PPM1D at Ser^1981^, which significantly downregulated ATM activity.^52^,^53^ PPM1D also targets proteins downstream of ATM. Chk2 is dephosphorylated by PPM1D at its DNA damage-induced, ATM-dependent phosphorylation site (Thr^68^), leading to decreased Chk2 kinase activity.^54^–^57^ PPM1D also dephosphorylates MDM2 at Ser^395^ and MDMX at Ser^403^ enhancing their stability, interaction, and the cooperative proteasomal degradation of p53.^58^–^60^ Although PPM1D has not been shown to target ATR directly, it does dephosphorylate downstream targets of ATR. We and others have demonstrated that PPM1D directly dephosphorylates Chk1 at its ATR-dependent phosphorylation site (Ser^345^), and in fact PPM1D, but not ATR, is important in regulating Chk1 phosphorylation in response to CDDP and ultimately chemosensitivity of ovarian carcinoma cells (Fig. 1B).^11^,^33^,^61^ PPM1D also dephosphorylates and inactivates γH2AX at Ser^139^, thereby inhibiting the formation of DNA damage foci, and recruitment and subsequent formation of DNA repair complexes.^62^–^64^ PPM1D has been shown to inhibit both base excision repair (BER) by dephosphorylating uracil DNA glycosylase (UNG2; Ser^6^)^34^,^65^ and nucleoside excision repair (NER) by dephosphorylating both excision repair proteins xeroderma pigmentosum complementation group A (XPA; Ser^196^) and complementation group C (XPC; Ser^892^).^66^,^67^

**Figure 2:**
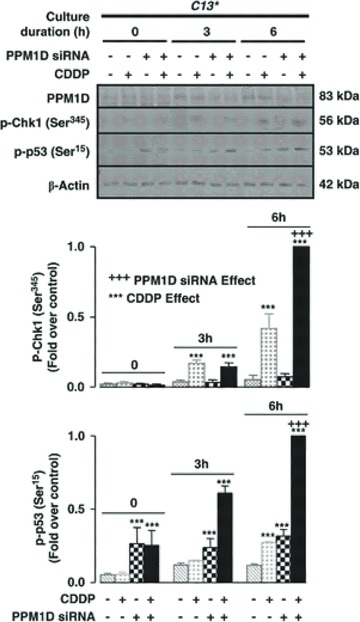
CDDP-induced, Chk1-mediated apoptosis is attenuated by PPM1D. PPM1D knockdown in C13* cells significantly upregulated p-Ser^345^-Chk1 and p-Ser^15^-p53 contents (*P* < 0.001). C13* cells were incubated with PPM1D siRNA or control siRNA (0–400 nM, 24 h), treated with CDDP (0–10 μM, 0–6 h), and PPM1D, p-Ser^345^-Chk1, p-Ser^15^-p53, and β-actin contents were assessed (*n*= 3). From Ref. 11.

PPM1D expression is induced by p53 and forms a negative feedback loop by dephosphorylating p53 at Ser^15^, a site important for its proapoptotic activity. We have demonstrated that PPM1D knockdown sensitizes resistant ovarian carcinoma cells to CDDP primarily by enhancing p53 activation via Ser^15^ phosphorylation (Fig. 1B and Fig. 2).^9^,^11^,^33^ However, the role of PPM1D in regulating p53 function goes beyond direct regulation, whereby PPM1D indirectly regulates p53 activation and stability. As mentioned previously, PPM1D regulates the activation of ATM, Chk1 and Chk2, known regulators of p53 activation, as well as MDM2 and MDMX, regulators of p53 stabilization. Moreover, PPM1D deactivates p38 mitogen-activated protein kinase (p38 MAPK) and downregulates the expression of its downstream effectors p16^Ink4a^ and p19^ARF^, which are vital tumor suppressors and important regulators of p53 activity.^32^,^51^,^68^ PPM1D ultimately inhibits DNA repair, cell cycle checkpoints, and cellular apoptosis, thereby promoting proliferation and passage of corrupted genome. By these means, PPM1D enhances oncogenic transformation and tumor growth.

PPM1D has two functional isoforms: full-length PPM1D (PPM1D605) and a shorter isoform, PPM1D430. PPM1D605 is ubiquitously expressed, while PPM1D430 is exclusively expressed in leukocytes and testes where it plays an important role in lymphocyte maturation and spermatogenesis, respectively.^69^ Both isoforms retain phosphatase activity and are able to dephosphorylate similar target proteins. PPM1D605 has two putative nuclear localization signal (NLS) domains and is assumed to be a strictly nuclear phosphatase, while PPM1D430 contains only one NLS and shows both nuclear and cytoplasmic localizations. However, we have found that PPM1D605 localization is differentially regulated between CDDP-sensitive and -resistant ovarian carcinoma cells, with PPM1D605 displaying cytoplasmic localization and nuclear exclusion in sensitive cells, and significant nuclear localization in resistant cells in response to CDDP (unpublished data).

## The PI3K/Akt survival pathway: regulation of PPM1D and p53

Another hallmark of cancer is the overexpression and/or activation of the phosphoinositide-3 kinase (PI3K)/Akt survival pathway. Improper activation of this pathway is associated with tumorigenesis in several tissue types.^70^–^74^ We have shown that Akt activation enhances the survival of ovarian carcinoma cells and promotes chemoresistance through attenuating p53 proapoptotic signaling.^2^,^4^,^5^,^7^,^9^

PI3K is a phospholipid kinase that phosphorylates the 3′ hydroxyl group of the inositol ring of phosphoinositide lipids. It is composed of a catalytic subunit (p110) and a regulatory subunit (p85).^75^ PI3K is activated in response to growth factors by several receptor tyrosine kinases (RTKs) that interact with the p85 subunit and activate the p110 catalytic subunit. Activated PI3K phosphorylates the membrane lipid phosphatidylinositol 4,5-bisphosphate (PIP_2_) to form phosphatidylinositol 3,4,5-triphosphate (PIP_3_), which recruits PI3K cytosolic effectors to the plasma membrane for further activation and downstream signaling. This process is regulated by the tumor suppressor phosphatase and tensin homolog (PTEN), which dephosphorylates PIP_3_ to PIP_2_, effectively shutting down the PI3K signaling cascade.^76^

One of the main downstream effectors of the PI3K pathway is the potent oncogenic serine/threonine kinase Akt, also known as protein kinase B (PKB). The Akt family consists of three members (Akt1, Akt2, and Akt3) that have high sequence homology and share similar domain structures, including an *N*-terminal pleckstrin homology (PH) domain necessary for protein–protein interaction, a central catalytic domain important for Akt kinase activity and a C-terminal regulatory domain.^77^ Akt is activated through recruitment to the plasma membrane by PIP_3_,^78^,^79^ followed by phosphorylation of Thr^308^ and Ser^473^ by the phosphoinositide-dependent kinase 1 (PDK1)^80^,^81^ and mammalian target of rapamycin complex 2 (mTORC2),^82^,^83^ respectively.

Activated Akt plays an important role in the regulation of several important molecular pathways, including cell survival, proliferation, and apoptosis. Akt phosphorylates the proapoptotic Bcl-2 family member BAD on its inhibitory Ser^136^ site, leading to decreased binding with Bcl-XL and, thus, inhibiting the release of mitochondrial death proteins.^84^ Akt also inhibits caspase-9 activity through phosphorylation of Ser^196^ (Ref. 85). Akt phosphorylates and inhibits the forkhead transcription factor 1 (FOXO-1) through phosphorylation of Thr^32^ and Ser^253^, facilitating its binding to 14–3-3 proteins and cytoplasmic sequestration, causing decreased expression of FOXO-1 targets, including the proapoptotic Bcl-2 family member Bim and Fas ligand.^86^ Akt activates the inhibitor of κB kinases (IKK) through phosphorylation of IKKα on Thr^23^, leading to IκB phosphorylation and degradation, and subsequent activation of the transcription factor NF-κB. NF-κB, in turn, promotes the transcription of several prosurvival genes, including PPM1D.^28^,^87^,^88^ Akt relieves cell cycle arrest at both the G_1_ and G_2_/M phases, resulting in increased proliferation. This is achieved through Akt-dependent phosphorylation and inhibition of p21 (Thr^145^) and downregulation of p27, which relieves the G_1_ checkpoint^89^–^91^ and Chk1 inhibitory phosphorylation (Ser^280^), inhibiting G_2_/M cell cycle arrest.^92^ Akt activates mTOR through direct phosphorylation at Ser^2448^, resulting in the activation of mRNA translational machinery.^93^ Akt also stabilizes the caspase-3 inhibitor X-linked inhibitor of apoptosis protein (XIAP) by phosphorylating Ser^87^, preventing XIAP auto-ubiquitination and subsequent proteasomal degradation.^94^ Akt regulates p53 protein stability and activation through phosphorylation of MDM2 on Ser^166^ and Ser^186^, facilitating its nuclear translocation and thereby enhancing p53 ubiquitination and proteasomal degradation.^95^,^96^

On the other hand, p53 can conversely regulate Akt activity by promoting the transcription of the PIP_3_ inhibitor PTEN, enhancing p53 protein level and activation as well as negatively regulating the expression of PI3K.^97^,^98^ We have observed that Akt plays an important role in the regulation of PPM1D protein stability and enhancement of its content in response to DNA damage in ovarian carcinoma cells. Overexpression of constitutively active Akt in chemosensitive ovarian carcinoma cells inhibited CDDP-induced PPM1D downregulation and significantly reduced CDDP sensitivity. PPM1D stability was enhanced following protein synthesis inhibition in resistant ovarian carcinoma cells, which contain constitutively active Akt, but not in their sensitive counterparts in response to CDDP treatment. Moreover, Akt downregulation in resistant cells leads to decreased PPM1D protein content in response to CDDP, but not mRNA expression. Furthermore, Akt downregulation in resistant cells significantly decreased PPM1D protein stability (unpublished data). Our observations suggest a possible new mechanism by which Akt can attenuate the activity of several tumor suppressors and disrupt the DNA damage response to enhance cellular survival, proliferation, and chemoresistance through positive regulation of its newly discovered effector, PPM1D.

## Targeting the PI3K/Akt pathway in ovarian cancer: the role of functional food compounds in tumor chemosensitization

Preventive medicine, when applied to the field of cancer research, predominantly focuses on the influence of lifestyle factors on carcinogenesis. While tobacco smoking and sedentary lifestyles as carcinogenic factors have attracted much recent attention, the importance of a diet high in fruits and vegetables has long been suspected to play a role in the prevention of multiple cancer types.^99^ Observations from epidemiological studies of dietary patterns have stimulated interest in the field of functional food research, which aims to characterize compounds from food sources that have health benefits beyond their normal nutritional properties. Various functional food compounds (FFCs) have been found to inhibit cancer growth or enhance the effects of treatment when combined with common chemotherapeutic agents.

A prominent example of an FFC is resveratrol, a phytoalexin found in red grapes and the focus of a 1997 landmark study into its antitumor activity.^100^ Evidence suggests that resveratrol inhibits numerous cellular factors that are involved in the initiation, promotion, and progression of cancer. Likewise, luteolin, a flavonoid present in cruciferous vegetables, including broccoli, inhibits protein kinase Cɛ and Src kinase activities, resulting in growth inhibition of UV-B induced skin cancer.^101^ Another well-established compound is curcumin, a natural phenol responsible for the yellow pigmentation of turmeric, which induces G_2_/M arrest and p53 activation, resulting in apoptotic responses in ovarian cancer cells.^102^

A potential application for FFCs is their use in cancer treatment to modulate signaling pathways involved in chemotherapeutic responsiveness. It has been demonstrated that FFCs, including resveratrol and curcumin, can enhance the effects of CDDP-insensitive tumors, thereby reducing the amount of cytotoxic agent required for a positive outcome.^103^,^104^ More importantly, various FFCs have been shown to induce chemosensitivity in resistant cells.^105^ This is especially critical for asymptomatic cancers that are often diagnosed late, such as ovarian cancer.

One aspect of chemoresistance can be seen as a failure of malignant cells to undergo apoptosis during chemotherapeutic challenge. This can arise from the dysregulation of multiple signaling pathways, biasing cell fate toward prosurvival decisions. Determinants of chemoresistance include p53 mutation, as well as the activation of cell survival intermediates and cascades such as FLIP, XIAP, and the PI3K/Ak pathway.^5^,^10^,^94^ Intervention targeting these mediators and their influences may therefore result in a cellular shift toward proapoptotic outcomes, emphasizing the possibility for FFCs exhibiting PI3K inhibitory ability to be employed in novel cancer treatment strategies. *In vitro* kinase assays using a number of FFCs from our laboratory indicate that piceatannol, hirsutenone, delphinidin, and cyanidin are potent inhibitors of PI3K (unpublished data). We have also demonstrated that piceatannol, a resveratrol analog metabolized by the cytochrome p450 enzyme CYP1B1, enhances the effects of CDDP in various ovarian cancer cell lines and inhibits various proteins implicated in cancer progression and chemoresistance. In addition, hirsutenone, which shares close structural similarity with curcumin, also displays potent chemosensitizing effects against CDDP-resistant ovarian cancer cells. These effects are notably increased if the cells contain a wild-type p53, suggesting that although p53-independent mechanisms of apoptosis can be triggered by FFCs, the presence of a functional p53 ensures a markedly higher response to treatment. If PPM1D is indeed regulated by Akt activity as our preliminary data suggests, it further strengthens the notion that specific inhibition of PI3K by a food compound has broad effects on p53-dependent chemosensitization as well as other, as yet unidentified, regulators of apoptosis.

## Conclusion

**Figure 3:**
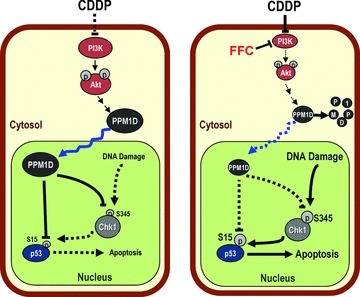
Hypothetical model illustrating the possible involvement of FFCs in Akt and PPM1D stability in ovarian cancer cells in response to CDDP treatment. In chemoresistant cells, activated Akt enhances PPM1D stabilization and nuclear import, subsequently suppressing Chk1 and p53 activation, and the induction of proapoptotic gene transcription. FFCs directly inhibit PI3K, leading to suppressed Akt activation and to PPM1D degradation. The loss of inhibitory action of PPM1D on nuclear p-Chk1 and p-p53 contents ultimately results in the induction of apoptosis and CDDP sensitization.

Ovarian cancer chemoresistance is a multifaceted conundrum and a better understanding of the molecular mechanisms involved will allow for the development of novel strategies for successful therapies. The cellular status of both Akt and p53, the interactions between them, and the net effect of these interactions will ultimately influence the outcome of treatment with chemotherapeutics. The discovery that PPM1D attenuates p53 activation and our own observations that Akt may stabilize PPM1D and enhance its content reveals a new mechanism by which Akt can regulate p53, besides the well-established Akt-MDM2 axis. However, our preliminary observations in relation to Akt-dependent PPM1D regulation requires additional validation and further experiments are underway to elucidate the complexity of this exciting relationship (Fig. 3).

Inhibition of the PI3K/Akt pathway is a logical step toward chemosensitization of resistant ovarian cancers, especially in tumors with a high PI3K/Akt activity profile. The use of FFCs in our lab has shown great promise in the sensitization of resistant ovarian cancer cells to CDDP-induced apoptosis. Their usage in combination with conventional chemotherapeutics holds a number of potential advantages over combinations with synthetic compounds. Their long history of human consumption can reasonably be expected to reduce the likelihood of adverse reactions arising from unknown toxicity or allergies. The screening of potential chemotherapeutics can also be less resource-consuming if a library is derived from food extracts, eliminating the need for complex biochemical synthesis of novel structures. Further understanding of chemoresistance pathways and how they are influenced by FFCs may lead to the development of novel cancer treatments that are less toxic, more affordable, and more effective.
